# Pediatric Oral Iron Therapy: Choosing the Right Product for Your Patient

**DOI:** 10.3390/hematolrep18020020

**Published:** 2026-03-05

**Authors:** Sonia Alexiadou, Emmanouela Tsouvala, Elpis Mantadakis

**Affiliations:** 1 Department of Pediatrics, University General Hospital of Alexandroupolis, 68100 Thrace, Greece; salexiad@med.duth.gr; 2 Neonatal Intensive Care Unit, University General Hospital of Alexandroupolis, 68100 Thrace, Greece

**Keywords:** ferrous sulfate, iron deficiency anemia (IDA), iron deficiency without anemia (IDWA), iron polymaltose complex, iron protein succinylate, liposomal iron, sucrosomial iron

## Abstract

In this narrative review, we address the prevention and therapy of iron deficiency anemia (IDA) with oral iron products in pediatric patients. Fortification of complementary foods with iron-containing micronutrient powders is the preferred method for the prevention of IDA in resource-limited settings. In developed countries, the prevention of sideropenia is through the consumption of iron-rich foods of animal origin. Regarding oral iron therapy, ferrous sulfate is the most widely used and cheapest product, but it is less well tolerated due to gastrointestinal side effects compared to complexes of ferric iron with polysaccharides, and complexes of iron with amino acids in casein, such as iron protein succinylate and iron acetyl aspartylate. These latter products are expensive and available only as single-dose vials with a fixed amount of elemental iron. Intermittent administration of ferrous sulfate, once or twice a week, is equally effective to daily therapy, with fewer side effects, and can be used in selected patients. Oral carbonyl iron has excellent bioavailability and the additional advantage of a high safety margin in cases of accidental overdose compared to iron salts, an important consideration given the potentially lethal consequences of iron overdose. Newer liposomal and sucrosomial iron products appear to have better intestinal tolerance and similar efficacy in the treatment of IDA, but limited pediatric data exist. In conclusion, all oral medicinal iron products are effective when prescribed for the treatment of IDA, if well-absorbed and taken consistently for 3 to 6 months. Physicians should be prepared to use alternative oral agents with better tolerance in case of gastrointestinal side effects.

## 1. Introduction

Anemia is a major public health problem, with the highest burden observed among young children, pregnant and postpartum women, and menstruating adolescent girls [1]. The World Health Organization (WHO) estimates that around 40% of children aged 6–59 months, corresponding to nearly 270 million children worldwide, are affected by anemia [2]. In developing countries, notably in sub-Saharan Africa, the prevalence of anemia in children of the same age is often >60% [3]. As dietary iron deficiency (ID) is the most common cause of anemia in children <5 years of age, iron deficiency anemia (IDA) represents the most prevalent form of anemia in children worldwide. In addition, ID without anemia (IDWA) is at least twice as common [4]. In the United States, the overall prevalence of sideropenia for toddlers 12–23 months and for non-pregnant adolescent girls aged 15–19 years old was 15.1% and 10.8%, respectively [5]. In a systematic review of European pediatric populations, which included 44 studies, ID prevalence in infants aged 6–12 months reached 2% to 25%, while in children aged 12 to 36 months it was between 3% and 48% [6]. Notably, the prevalence of IDA was typically <5% in Northern and Western Europe but increased markedly in Eastern European countries, reaching levels of up to 40–50% in young children [6].

Hepcidin, a peptide hormone produced by the liver, is the master regulator of iron homeostasis by controlling the intestinal absorption of iron, its recycling within the cells of the reticuloendothelial system, and its storage. It acts by binding to the transmembrane protein ferroportin, the only known cellular iron exporter, causing its internalization and degradation [7,8]. High serum hepcidin levels induced by inflammation block iron absorption, whereas low hepcidin levels, as typically seen in children with IDA due to nutritional reasons, allow increased iron absorption.

## 2. Risk Factors for IDWA and IDA

Diets with low iron bioavailability are the main contributor to IDA in developing countries, where access to iron-rich meat-based foods is limited by high cost and availability [9]. In developed countries, the leading causes of IDWA and IDA in children are inappropriate feeding practices, as well as chronic blood loss from the gut or the genitourinary system [10]. Regarding infants and young children, inappropriate feeding practices include prolonged exclusive breastfeeding without iron supplementation, introduction of cow’s milk before the first birthday, extreme cow’s milk intake (>24 ounces daily), and continued bottle use beyond the first birthday [5,11]. Other risk factors include prematurity, as birth iron stores are important in the prevention of early ID, low iron diet, obesity [12], heavy menstrual bleeding, inflammatory bowel diseases [13], and low socioeconomic status.

Early diagnosis and appropriate management of sideropenia are of paramount importance, given the critical role of iron in the development of the central nervous system in children and evidence that IDWA/IDA in infancy may be associated with lasting neurocognitive effects, adverse psychomotor plus socioemotional development, even after correction of the deficiency [14,15,16].

## 3. Prevention of IDWA and IDA

In settings where anemia prevalence among children <5 years exceeds 20%, the WHO recommends adding iron-containing micronutrient powders (MNPs) for fortification of complementary foods in infants and young children aged 6 to 23 months [17]. In these formulations, iron is encapsulated within a lipid coating to minimize adverse effects (AEs) on the taste and appearance of foods. MNPs are provided in single-dose sachets and are added to the child’s meal after serving. The MNPs typically contain 12.5 mg of elemental iron per sachet, which corresponds to 37.5 mg of ferrous fumarate or 62.5 mg of ferrous sulfate (FeSO_4_) heptahydrate, or equivalent amounts of other iron compounds [18]. De-Regil et al. assessed the effects of point-of-use fortification of foods with iron-containing MNPs alone, or in combination with other vitamins and minerals, on nutrition, health, and development among preschoolers and children of school age, compared with no intervention, a placebo, or iron-containing supplements. They reviewed 13 studies involving 5810 participants from Latin America, Africa, and Asia. MNPs containing iron were successful in reducing anemia and IDWA in preschool- and school-age children. However, information on developmental outcomes and AEs was very limited [19]. The American Academy of Pediatrics (AAP) recommends supplementation of preterm neonates with 2 mg/kg/day of enteral iron, either as medicinal iron or in the form of iron-fortified formula. It is recommended that this start at one month of age and is continued until the first birthday. It is also recommended that full-term infants who are exclusively breastfed, as well as those who receive more than half of their daily nutrition from human milk and are not yet consuming iron-rich complementary foods, receive an iron supplement of 1 mg/kg per day, starting at 4 months of age and continued until appropriate iron-containing complementary foods have been introduced [20].

Preventive oral iron supplementation is one of the strategies used to reduce the burden of IDWA/IDA in populations with high anemia prevalence (≥40%). According to the WHO, infants and young children aged 6–23 months should receive daily oral iron supplementation at doses of 10–12.5 mg administered for three consecutive months each year [21].

## 4. Medicinal Products of Oral Iron

There are numerous oral iron formulations available in various dosage forms suitable for pediatric use, including drops, syrups, sublingual sprays, single-dose vials, capsules, and tablets. Many of these preparations belong to the category of ferrous salts, of which FeSO_4_ is the most commonly used and extensively studied, and which is considered the standard of care. Other types of oral iron include ferric (Fe^3+^) iron complexes with polysaccharides, complexes of iron with amino acids in casein, including iron protein succinylate and iron acetyl aspartylate, as well as sucrosomial iron, carbonyl iron (CI), and heme iron polypeptides. Not all products are available in every market, but their large number is indicative of the absence of an ideal oral iron product. Table 1 summarizes commercially available oral iron products.

Oral iron is usually the first line of therapy in children with IDA due to the convenience and low cost of most oral formulations. Published evidence suggests that daily oral supplementation of iron alone, in combination with folic acid, or other vitamins or minerals, or both, may result in a significant reduction in IDA across various populations, with stool discoloration being the most common side effect [22]. Parenteral iron therapy should be used only when oral therapy has failed or when a quick resolution of the IDA is required. Oral iron therapy is preferred for the treatment of IDA/IDWA because it does not require hospitalization and is usually effective, provided that there is an intact gut and good compliance with the administered therapy. Moreover, oral therapy does not carry the risk of a serious, albeit extremely rare, anaphylactic reaction that can occur with any parenteral iron product [23]. If a rapid resolution of moderate-to-severe IDA is required, parenteral iron therapy is preferable because it corrects sideropenia more quickly and with only one or a few intravenous infusions, provided that newer third-generation IV iron products are used (ferric carboxymaltose, ferric derisomaltose) [24]. For example, in symptomatic adolescent girls with moderate to severe IDA, we use intravenous iron, like ferric carboxymaltose, upfront because it corrects the anemia faster, and when the bleeding is fully controlled, and the patient has recovered, we recommend prophylactic oral iron therapy on the 4–5 bleeding days of the menstrual cycle. In addition, we recommend the use of oral contraceptives to regulate blood flow and/or oral anti-fibrinolytic products, like oral tranexamic acid, to minimize blood loss during menstrual periods [25]. Table 2 contrasts oral and intravenous iron therapy. For this manuscript, we will focus on oral iron therapy alone. Although we review mostly pediatric studies, some adult clinical trials are also mentioned when pediatric data are scarce. We will not talk about ferric maltol, sodium ferric ethylenediaminetetraacetate, and heme iron polypeptide because these products are not commercially available in Greece.

## 5. Oral Iron Therapy

Iron salts are frequently administered for the prevention and treatment of sideropenia, in spite of their relatively poor bioavailability and frequent AEs. Their use is frequently associated with gastrointestinal AEs, which may compromise adherence to therapy. These include nausea, vomiting, epigastric discomfort, abdominal pain, constipation, diarrhea, black stools, teeth staining [26], and an unpleasant metallic aftertaste. Regarding the latter, in a prospective study to characterize obstacles and enhancers of oral iron therapy in children aged 9 months to 4 years with IDA, poor taste was identified by the parents as a major barrier [27]. Although hemoglobin levels often respond rapidly to patients who adhere successfully to treatment, oral iron therapy requires 3 to 6 months to achieve resolution of the anemia, normalization of serum ferritin levels, and replenishment of body iron stores [28]. An appropriate response to oral iron is demonstrated by reticulocytosis within 5 to 7 days, followed by an increment in hemoglobin within the first 1 to 2 weeks from initiation [29]. An optimal response to oral iron therapy is typically defined by an increase in hemoglobin concentration of approximately 2 g/dL within three weeks [30]; however, increase of 1 g/dL within 4 weeks is considered reasonable [31]. Serum ferritin should be interpreted along with serum C-reactive protein (CRP) [32].

An ideal oral iron formulation should combine efficacy with optimal tolerability and ease of use, particularly in children. It should ideally be administered once daily or, at most, in two daily doses to improve compliance. Easy swallowing, an acceptable taste without a metallic aftertaste, and the absence of dental staining are important additional practical considerations. High bioavailability that is not diminished by food intake is highly desirable, along with a favorable safety profile characterized by minimal gastrointestinal side effects. In addition, the ideal oral iron formulation should have a long shelf life at room temperature after opening and remain affordable. Finally, minimal toxicity in the event of accidental overdose represents a critical safety feature, especially in children. Iron remains one of the most common causes of pediatric deaths reported to poison control centers [33]. Juurlink et al. demonstrated that 42% of iron poisonings occurred within one year of the birth of a sibling. In addition, they found a fourfold increase in the risk of iron poisoning in patients whose mothers were in the first postpartum month, a period when prenatal vitamins are more likely to be found at home [34]. Table 3 summarizes the desirable properties of an ideal medicinal oral iron product.

Current treatment recommendations for iron-deficient children are not firmly evidence-based, and the recommended oral iron dose of 3–6 mg/kg/day is primarily based on empirical practice [28]. Clinical trials in children are relatively scarce and characterized by substantial heterogeneity in terms of iron formulations used, dosing strategies, and treatment duration. This lack of consistency and standardization significantly limits the comparability of findings and frequently complicates therapeutic decision-making. The practice of administering oral iron in two or three divided daily doses is being increasingly replaced by once-daily dosing, as studies have shown comparable therapeutic efficacy with improved compliance and lower costs [28]. Zlotkin et al. performed a randomized controlled clinical trial (RCT) in 557 anemic children. One group received FeSO_4_ drops once daily, and the other received FeSO_4_ drops tid per day (total dose, 40 mg of elemental iron for both groups). They showed that, over a two-month period, once daily and tid dosing achieved comparable results for IDA with no reported adverse effects [35]. To reduce the burden of IDA, intermittent iron supplementation regimens have been studied. Investigators from Pakistan compared the results of once-weekly vs. daily oral iron supplementation in children of school age. For 2 months, FeSO_4_ 200 mg was given daily to the first and once weekly to the second group. A significant and comparable improvement in hematologic parameters was observed in both groups. Moreover, weekly iron supplementation had few, if any, side effects [36]. Tavil et al. randomized 94 children between the ages of 5 months and 6 years with IDA to receive FeSO_4_ at 6 mg/kg daily or the same dose biweekly. Both treatment groups were reevaluated for hematological response at 2 months. Intermittent treatment was equally effective but better tolerated than daily treatment [37]. In a study from Jordan, 134 children, 2 to 6 years old, were randomly assigned to receive daily, weekly, or biweekly 5 mg/kg of elemental iron for three months as FeSO_4_ drops along with parental nutritional counseling. Weekly and biweekly oral iron therapy was equally effective as daily oral iron therapy in correcting IDA, as indicated by a similar rise in hemoglobin [38]. In a systematic review and meta-analysis of 129 RCTs providing ≥30 days of oral iron supplementation versus placebo or control to children and adolescents aged <20 years, the results suggest that frequent (3–7 times/week) and intermittent (1–2 times/week) iron supplementation are equally effective at decreasing IDWA and IDA [39]. Nevertheless, most available data relate to formulations of ferrous salts, which were used in approximately 70% of the included studies (FeSO_4_ 60.7%, ferrous fumarate 10%) [39]. Consequently, the application of intermittent dosing regimens with other iron formulations should be approached with caution, as supporting evidence remains limited.

### 5.1. Ferrous Sulfate

FeSO_4_ is available in syrup, tablets, and capsules. For better oral absorption, it is administered 30 min to two hours before or after meals along with orange juice, as an iron absorption facilitator [40]. Administration with food decreases bioavailability and thus should be consumed on an empty stomach. Twenty-one toddlers with mild to moderate IDA aged 6 to 17 months received an oral FeSO_4_ heptahydrate solution at 2 mg/kg of elemental iron daily, and 19 of them were analyzed for hematologic response at three months. Hemoglobin and ferritin were normalized in 95% and 84% of the patients, respectively. Only one patient experienced upper abdominal pain [41]. An Indian study randomly assessed the clinical response and AEs of FeSO_4_ and iron polymaltose complex (IPC) in 118 children with IDA. All participants were given elemental iron in three divided doses at 6 mg/kg/day, 30 min before meals, for 30 days. Children who received FeSO_4_ had higher hemoglobin and fewer residual complaints at 30 days as compared to those who received IPC. However, gastrointestinal side effects were 2.5 times more common in those who received FeSO_4_ [42]. Powers et al. randomized infants and children 9 to 48 months with IDA to receive low-dose (3 mg/kg of elemental iron) FeSO_4_ or IPC once daily for 12 weeks. From baseline to the end of the study, mean hemoglobin increased from 7.9 to 11.9 g/dL (FeSO_4_ group) vs. 7.7 to 11.1 g/dL (IPC group), a greater difference of 1.0 g/dL (*p* < 0.001). Median serum ferritin level increased from 3 to 15.6 ng/mL (FeSO_4_) vs. 2.0 to 7.5 ng/mL (IPC), a greater difference of 10.2 ng/mL (*p* < 0.001) [43]. In an important Indonesian study, 50 infants, 12 to 18 months old with IDA, were randomly assigned to receive oral FeSO_4_ 3 mg/kg/day or a placebo for 4 months. Similar treatment randomization was done among 29 infants with IDWA and 47 iron-sufficient infants. All infants underwent mental and motor assessment in the Bayley scales before enrollment and after intervention. Developmental delays were significantly more common among infants with IDA, and treatment with FeSO_4_ reversed them, having no significant effects on the scores of the infants with IDWA and on the iron-sufficient infants. It should be noted that this study was underpowered to answer the question of whether IDWA affects mental and motor development [44]. Parkin et al. conducted a blinded, randomized, placebo-controlled clinical trial in 8 primary care practices in Toronto, Canada, comparing the effects of ferrous sulfate or a placebo on neurodevelopmental and laboratory outcomes in children 1–3 years old with IDWA (hemoglobin >11 g/dL, serum ferritin <14 ng/mL). The outcomes of interest were the Early Learning Composite (ELC) using the Mullen Scales of Early Learning, and the serum ferritin at 4 and 12 months. For ELC, the mean between-group difference was uncertain. However, for serum ferritin, at 4 months, the mean between-group difference was 16.9 ng/mL. Moreover, the mean serum ferritin increased substantially from baseline to 12 months from 10 to 22.8 ng/mL, clearly indicating that complementing ferrous sulfate to diet advice resulted in superior serum ferritin [45]. In a systematic review of 111 studies on oral iron therapy, which included data on 10,695 patients, extended-release FeSO_4_ with mucoproteose had the lowest incidence of AEs (4.1% overall, 3.7% for gastrointestinal AEs). Incidence rates of overall AEs for the other supplements were 7.3% for iron protein succinylate, 23.5% for ferrous glycine sulfate, 30.9% for ferrous gluconate, 32.3% for FeSO_4_ without mucoproteose, and 47% for ferrous fumarate. The differences in incidence of AEs between extended-release FeSO_4_ with mucoproteose and all other supplements except iron protein succinylate were statistically significant [46].

### 5.2. Ferrous Ascorbate

Ferrous ascorbate is considered to have better tolerance compared to FeSO_4_ [47]. Patil et al. performed an RCT on 125 children aged 1 to 12 years with IDA. Both groups randomly received ferrous ascorbate or IPC at 6 mg/kg/day of elemental iron for 3 months. Both iron products used produced statistically significant improvements in the hematological parameters during the 3 months of intervention, but the improvement was significantly better with ferrous ascorbate [48]. Yewale and Dewan compared ferrous ascorbate and colloidal iron in the treatment of IDA in 73 children. The provided iron dose corresponded to elemental iron 3 mg/kg/day. The mean rise in hemoglobin at 12 weeks was significantly higher in the ferrous ascorbate group [3.59 ± 1.67 g/dL vs. 2.43 ± 1.73 g/dL; *p* < 0.01] [49].

### 5.3. Ferrous Fumarate

Ferrous fumarate demonstrates absorption comparable to that of FeSO_4_ in non-anemic, iron-sufficient infants and young children, and can be recommended as an effective fortificant for complementary foods designed to prevent ID [50]. The WHO recommended regimen for the prevention of IDA is one sachet of MNP powder daily that contains 12 mg iron as encapsulated ferrous fumarate [17]. A screen-and-treat plan based on hepcidin of targeting iron administration in children aged 6 to 23 months accomplished a reduction in the overall dose of iron used, but was substantially less effective in combating IDA than the abovementioned WHO’s standard of care [51]. Zlotkin et al. showed that the daily use of a single dose of microencapsulated ferrous fumarate sprinkles that contained ascorbic acid was equally effective to FeSO_4_ drops in the management of IDA in anemic children aged 6 to 18 months without side effects [52]. Grover et al. performed an RCT in 40 children aged 6 months to 10 years with IDA. The first group received oral ferrous fumarate at 4 mg/kg in two divided doses daily. The second received the same dose on alternate days. Serum hepcidin was measured at baseline and 48 h after initiation of therapy, while hemoglobin was recorded after one month of therapy. Children on alternate-day therapy had higher hemoglobin on day 30, decreased serum hepcidin at 48 h, and fewer gastrointestinal side effects [53]. Tchum et al. performed a population-based, randomized cluster trial of longstanding prophylactic iron fortification on the probability of IDA in preschool children living in a malaria-endemic area. The intervention group received daily 12.5 mg of elemental iron, as ferrous fumarate, along with vitamin A, ascorbic acid, and zinc for 5 months. The placebo group received a similar MNP powder but without iron. The two groups had similar baseline anthropometric, demographic, dietary, and clinical characteristics. Of the 1904 children who remained in the study, the intervention group had significantly higher hemoglobin and serum ferritin than the placebo group [54]. However, in another study, iron absorption from ferrous fumarate was considerably lower than that of FeSO_4_ in children 2 to 5 years old with IDA, questioning the preventive efficacy of ferrous fumarate in iron fortification programs [55].

### 5.4. Iron Polymaltose Complex

Ferric iron in the form of iron-hydroxide polymaltose complex has been shown in several clinical trials in infants, children, and adults to be effective in treating IDA [56]. Due to its pharmacokinetic properties, IPC is best administered with meals and in higher doses than those of classical ferrous iron salts, such as FeSO_4_. Many studies have shown a lower rate of treatment interruption with IPC than with ferrous salts due to a lower incidence of AEs from the upper gastrointestinal tract [57]. Jaber et al. in Israel prospectively compared the efficacy and safety of sideropenia prophylaxis with iron gluconate (IG) or IPC in 105 healthy infants who attended a pediatric community center. Children were randomized to consume one of the two iron formulations starting at the age of 4 to 6 months until their first birthday. Mean hemoglobin at 12 months was substantially better in the IG group. However, gastrointestinal AEs were considerably less common in the IPC group [58].

### 5.5. Carbonyl Iron

A Brazilian study evaluated the efficacy of chewable CI tablets for the treatment of IDA in children <6 years old compared to FeSO_4_. Seventy-three children were recruited. Participants were assigned to receive either chewable CI tablets or a solution of FeSO_4_ for 90 days, both at a dose of 5 mg/kg/day. After 30 days of treatment, hemoglobin increased by 1.3 g/dL and by 1.2 g/dL in the CI and FeSO_4_ group, respectively. By the end of treatment, those who received CI again had significantly higher hematocrit and serum ferritin compared to the FeSO_4_ group [59]. In a 3-week double-blind RCT involving 36 female blood donors with mild ID, high-dose CI (600 mg tid) was well tolerated compared to FeSO_4_ 60 mg tid [60]. The 10-fold larger amount of CI resulted in a mean 1.5-fold increase in estimated iron absorption with similar gastrointestinal side effects. The major advantage of CI is its noticeably greater margin of safety when compared to FeSO_4_. In humans, the estimated lethal dose of oral FeSO_4_ is 200 mg Fe/kg body weight. On the other hand, human volunteers have taken doses of 10,000 mg of CI with no side effects [60]. Finally, no known case of corrosive gut injury from the consumption of CI has ever been reported to date [61].

### 5.6. Ferrous Bisglycinate

Ferrous bisglycinate is an amino acid iron chelate that is considered more bioavailable and with fewer gastrointestinal AEs as compared with iron salts. A meta-analysis of 17 RCTs that reported hemoglobin or ferritin following at least 4 weeks’ supplementation of ferrous bisglycinate, compared with other iron supplements, showed a non-significant trend for higher ferritin concentrations in pregnant women. Regarding children, no significant differences in hemoglobin or ferritin concentrations were reported. Hence, more trials are needed to assess its efficacy in children compared to cheaper iron salts [62]. Giancotti et al. tested the efficacy of a new oral iron supplement combining ferrous bisglycinate chelate with sodium alginate in patients with celiac disease, an immunologically mediated disorder characterized by iron malabsorption. Twenty-six adults affected by IDA, of whom 14 were also affected by celiac disease, were enrolled. An oral iron absorption test was performed in each patient by administering the new oral iron supplement. Therapy was well tolerated, and a similar improvement in serum iron occurred in patients with IDA plus celiac disease versus patients with isolated IDA [63]. The Associazione Itaiana di Ematologia ed. Oncologia Pediatrica prospectively recorded oral iron therapy in children, aged 3 months to 12 years with IDA, who were treated in one of 15 Italian centers. Of the 107 analyzed patients, 67 received ferrous bisglycinate iron 0.45 mg/kg, 18 received ferrous gluconate/sulfate 2 mg/kg (ferrous 2), 7 ferrous gluconate/sulfate 4 mg/kg (ferrous 4), 7 ferric iron salts 2 mg/kg, and 13 liposomal iron 0.7–1.4 mg/kg. A higher median increase was noted in the ferrous 2 and 4 groups at 2 and 8 weeks. Gastrointestinal adverse effects occurred in 16%, 14%, 6% and 0% of ferrous 2, ferrous 4, ferrous bislycinate, and ferric and liposomal iron patients, respectively. Hence, bisglycinate iron appears to have an excellent safety profile, but may be less effective than ferrous salts in rapidly increasing the hemoglobin of children with IDA [64].

### 5.7. Hydrogen-Reduced Iron (HR Fe)

At high temperatures, hydrogen gas reduces iron oxide into elemental iron powder, resulting in a pure, fine powder. This product, known as hydrogen-reduced iron (HR Fe), is used for public health fortification in cereal or other basic foods like flour to combat IDA, with low cost, and without affecting taste or color. However, its bioavailability is substantially lower than that of ferrous salts. Still, it can be improved if prepared into fine particles <45 µm, a process known as micronization, or if added post-baking [65].

### 5.8. Iron Protein Succinylate

Iron protein succinylate contains ferric iron complexed with succinylated casein protein to form a compound with high-molecular-weight and improved oral bioavailability [66]. It resists stomach breakdown, allowing its intestinal dissolution, where iron release and absorption take place. In a study from Greece, 100 children, aged 1 to 9.4 years with IDWA or IDA, were randomly assigned to receive 4 mg/kg of elemental iron with a maximum daily dose of 80 mg daily, for 2 months, as either iron succinylate or IPC. Side effects and efficacy were assessed after 30 and 60 days of therapy. Both drugs were well tolerated. Iron protein succinylate led to a faster increase in hemoglobin and ferritin than IPC, which was sustained after 2 months of treatment [67]. In a systematic review of iron protein succinylate that included 54 studies with a total of 8454 subjects, including 960 children (premature infants to 14-year-old children), patients who received iron protein succinylate had the lowest rate of AEs, proving it is a valuable and tolerable treatment option for IDWA and IDA [66]. However, due to its high cost, it should be used when cheaper first-line ferrous salts or IPC products have failed.

### 5.9. Liposomal Iron

In liposomal iron, ferric ions are encapsulated into liposomes. This bypasses the hepcidin–ferroportin axis, leading to absorption through the intestinal M-cells. Investigators from Egypt completed a prospective RCT in 192 children with IDA. Patients received liposomal iron (1.4 mg/kg/day) or ferric iron as IPC (6 mg/kg/day). Both medicinal irons were administered once daily. After one month of therapy, children receiving liposomal iron showed higher hemoglobin and serum ferritin levels. After 6 months of therapy, the hemoglobin and growth-related anthropometric measurements were significantly higher in patients who received liposomal iron. Moreover, liposomal iron use was associated with improved patient compliance [68]. Sharma et al. compared the effects of oral liposomal iron with those of FeSO_4_ as intermittent prophylaxis against IDA in children aged 6 to 59 months. Forty children in each group received 20 mg of elemental iron twice weekly for 3 months. The hemoglobin and biochemical markers of sideropenia showed greater improvement in those who received liposomal iron, who also experienced fewer side effects and better compliance [69]. In a very recently published study, 433 children aged 6 months to 5 years were tested for IDA and IDWA. The group without anemia was divided into one subgroup that received a placebo and another that received liposomal iron for four months. Patients with IDA also received liposomal iron for four months. Hematologic efficacy and tolerability, development, and growth were evaluated before and after treatment. The interventional group with IDWA improved substantially with regard to the total developmental scores compared to the placebo group. The final score was better in the isolated sideropenia group that took liposomal iron compared to the IDA group (*p* < 0.001). Hence, liposomal iron is efficacious, tolerable, and improves the development and growth of iron-deficient children, with the best results obtained from early intervention before the establishment of anemia [70]. In another recent study, 60 pediatric patients with chronic kidney disease (CKD) were randomized to receive oral liposomal iron 30 mg/day or intravenous iron dextran 50 mg tiw for 3 months. Daily liposomal iron was equally or more effective than intravenous iron dextran for the treatment of these anemic children with CKD, with no reported AEs during the study period [71].

### 5.10. Sucrosomial Iron

Sucrosomial iron is a novel oral iron formulation in which ferric pyrophosphate is protected by a phospholipid bilayer, mainly from sunflower lecithin, plus a sucrester matrix (sucrosome), which is absorbed through paracellular and transcellular routes (M cells) [72]. In adults, high-dose oral sucrosomial iron (60 mg *per os* bid) was equally effective to intravenous ferric gluconate in patients refractory/intolerant to oral FeSO_4_ [73]. Suva and Tirgar, in a retrospective observational study of 260 adults with IDA, showed that sucrosomial iron, compared to ferrous fumarate, ferrous ascorbate, and ferrous bisglycinate, was more effective in improving hemoglobin and iron indices and with a better safety profile [74]. In a single-center, retrospective, observational cohort study of children with newly diagnosed IBD, treatment with sucrosomial iron was effective in 88% of the patients at the end of follow-up, regardless of IDA severity at baseline, and with no serious AEs [75]. The same conclusion was reached in a multi-center study in 52 adults with IBD-IDA, where 30 mg of sucrosomial iron daily for 12 weeks led to improved IDA symptoms, IBD activity, and patients’ quality of life, with excellent adherence to the study medication [76]. However, in a cross-sectional, retrospective Turkish study, IDWA/IDA was observed at a significantly higher rate in children aged 9 to 13 months using sucrosomial and microencapsulated iron prophylaxis compared to children who received conventional ferrous or ferric iron salts [77].

One of the reasons physicians are not familiar with the newer oral iron products and continue to use ferrous iron salts and iron polymaltose complex is that these older formulations are typically reimbursed by National Health Systems, which is not the case with many of the newer products that are frequently marketed as over-the-counter dietary supplements. The need for out-of-pocket expenses is a reason to defer the use of newer oral iron products.

## 6. Use of Oral Iron for Prevention and Treatment of IDA in Neonates

Iron supplementation in neonates should follow the guidelines of the AAP mentioned above [20]. Formula-fed infants up to the age of 6 months should consume an iron-fortified infant formula with 4–8 mg/L of elemental iron [11]. Infants weighing 2000–2500 g should receive daily iron supplements of 1–2 mg/kg. ESPHAGAN recommends the use of colloidal iron [78].

For premature and other high-risk infants at risk for IDWA/IDA, we recommend closely monitoring serum ferritin levels and supplementing iron up to 15 mg/kg/day to maintain iron adequacy [79]. Among ferrous iron salts, FeSO_4_ and iron gluconate have been more extensively used in neonates; IPC and ferrous bisglycinate have also been successfully used, while studies are underway to assess the efficacy and safety of liposomal and sucrosomial iron in neonates.

Based on a systematic review of 27 articles, including 18 RCTs, long-term iron supplementation results in a reduction in IDA in preterm and low-birth-weight (LBW) infants [80]. Franz et al. randomly assigned very-low-birth-weight (VLBW) infants <1300 g to receive FeSO_4_ 2 to 6 mg/kg/day when enteral feedings of >100 mL/kg/day were established (early group) or at 61 days of life (late group). The primary outcome was ferritin and the number of infants with ID. Ferritin at 61 days was similar between the two groups. However, more blood transfusions were required after day 14 of life in infants of the late group [81]. Supplementing VLBW infants with colloidal ferric hydroxide 3 mg/kg for infants 1000–1500 g and 4 mg/kg for infants < 1000 g starting at 2 weeks of age did not improve serum ferritin or hematological parameters at 2 months when compared to the standard practice of starting iron at 2 months [82]. However, a similar RCT with the same iron product at a dose of 2 mg/kg/day showed the opposite, with the early iron intervention at 2 weeks leading to improved serum ferritin and hemoglobin [83]. This study is in agreement with Arnon et al., who randomized infants <32 weeks’ gestational age to early or late iron supplementation (2 vs. 4 weeks) with IPC and showed that iron status was better in the early than in the late group [84].

## 7. Conclusions

In conclusion, the commercially available oral iron products are numerous and have different pharmacokinetic properties. Ferrous iron salts have better absorption than ferric iron products, at the expense of a higher rate of gastrointestinal side effects, when given in similar doses of elemental iron. In our clinical practice, we prefer to use FeSO_4_ therapeutically up to 3 mg/kg once daily to improve gut tolerance. In selected patients without persistent risk factors for IDA, such as ongoing blood loss or consumption of vegetarian/vegan diets with low iron content, intermittent therapy with once or twice weekly FeSO_4_ appears to be equally effective to daily administration, and with substantially lower AEs. In case of intolerance, IPC, ferrous bisglycinate, and CI are acceptable choices. In young infants who feed every 2 to 3 h, we prefer to use IPC drops or syrup, because its absorption is enhanced by food consumption but at higher doses compared to FeSO_4_, i.e., 6 mg/kg/day, due to decreased bioavailability. For adolescents with IDA, we use tablets or slow-release capsules of FeSO_4_ with mucoproteose. In case of intolerance, iron protein succinylate, at a dose of 40 to 80 mg of elemental iron daily, and liposomal or sucrosomial iron at doses up to 30 mg once or twice daily are appropriate therapeutic choices. Finally, for successful treatment of IDA/IDWA, iron supplementation requires individualized, patient-specific approaches, for which practitioners should familiarize themselves with a few oral iron products from the numerous options available to quickly and effectively treat patients in need.

## Figures and Tables

**Table 1:** Commercially available oral iron products. Not all products are available in every market.

**Ferrous Iron Salts**	Ferrous Sulfate (Gold Standard), Ferrous Gluconate, Ferrous Fumarate, Ferrous Acetate, Ferrous Ascorbate. Available in Many Liquid and Edible Oral Forms.
**Ferric iron salts**	Ferric citrate (used mostly as an oral phosphate binder in patients with chronic kidney disease). Ferric Na_2_EDTA (sodium feredetate).
**Heme iron polypeptides**	Available in many liquid and edible oral forms.
**Carbonyl iron**	Popular in the USA as stand-alone tablets or in combination with zinc, folic acid, vitamin C, and vitamin B12 in various combinations. Available as an oral suspension with MCT oil in some countries.
**Ferrous glycine chelates (compounds of chelated iron with glycine)**	Ferrous bisglycine chelate, ferric trisglycine chelate, ferrous bisglycinate hydrochloride, ferric glycinate
**Ferric hydroxide polymaltose complex (iron polymaltose complex, IPC), ferric maltol**	Available in drops, syrup, chewable tablets.
**Iron succinylate**	Complex of 5% ferric iron (Fe^3+^) with succinylated casein or acetyl-aspartyl-casein.
**Liposomal iron**	Ferric pyrophosphate encapsulated in phospholipid vesicles.
**Sucrosomial iron**	Ferric pyrophosphate encapsulated within sucrosomes. It contains ascorbic acid (vitamin C) as a facilitator of iron absorption. The sucrosome is made by pregelatinized rice starch, sucrose esters of fatty acids, sunflower lecithin, glucose syrup, tricalcium phosphate, and milk proteins.

**Table 2:** Comparison of oral and intravenous iron therapy for IDA in children.

	Oral Iron Therapy	Intravenous Iron Therapy
**Cost**	Low, but products of iron protein succinylate, liposomal, and sucrosomial iron can be expensive	High, but the limited need for laboratory work-up post-therapy reduces the total cost
**Need for hospitalization**	No	Yes, but with 3rd generation products, single or a few short IV infusions are required
**Speed of anemia resolution**	Slow	Fast
**Gastrointestinal side effects**	Relatively common but depend on the product and the dose chosen. Ferric iron products are better tolerated	Absent
**Risk of anaphylaxis**	No	Minimal with 2nd and 3rd generation IV iron products
**Need for frequent laboratory work-up to check for response**	Yes	No

**Table 3:** Desirable properties of an ideal oral iron product.

Available in Both Liquid and Edible Forms
Easy swallowing
Good taste, no metallic aftertaste
No or minimal discoloration of the teeth
Excellent bioavailability, unaffected by the type of food consumed
Easy administration schedule (once or twice daily)
Long shelf life after opening
Does not require a refrigerator for safe preservation
Low cost
No or minimal gastrointestinal side effects
Minimal systemic toxicity in case of overdose

## Data Availability

No new data were created or analyzed in this study. Data sharing is not applicable to this article.

## Abbreviations

The following abbreviations are used in this manuscript:

AAP	American Academy of Pediatrics
AEs	Adverse Effects
bid	Twice a day
CI	Carbonyl Iron
CKD	Chronic Kidney Disease
CRP	C-reactive protein
ESPHAGAN	European Society for Paediatric Gastroenterology Hepatology and Nutrition
FeSO_4_	Ferrous Sulfate
IBD	Inflammatory Bowel Disease
ID	Iron Deficiency
IDA	Iron Deficiency Anemia
IDWA	Iron Deficiency Without Anemia
IPC	Iron Polymaltose Complex
LBW	Low Birth Weight
MCT	Medium-chain triglycerides
MNPs	Micronutrient Powders
RCT	Randomized Controlled Trial
tiw	Three times a week
VLBW	Very Low Birth Weight
WHO	World Health Organization
